# Is primary care the ‘right care’ for respiratory medicine in Africa?

**DOI:** 10.4102/phcfm.v18i1.5659

**Published:** 2026-08-29

**Authors:** Robert J. Mash

**Affiliations:** 1 Division of Family Medicine and Primary Care, Faculty of Medicine and Health Sciences, Stellenbosch University, Cape Town, South Africa

The International Primary Care Respiratory Group (IPCRG) just finished their 25th anniversary global conference in Tunisia. One of its campaigns is for Asthma Right Care:

[*D*]oing the right things and only the right things in the right way for the right people at the right time in the right place, whatever that means in the local context.^1^

They are convinced that primary care is central to the delivery of right care for people with asthma and other respiratory conditions. But is this true in the African context?

The World Health Organization (WHO) is clearly committed to ‘advancing the primary health care approach and essential system capacities for universal health coverage’.^2^ The United Nations General Assembly has also made recommendations on non-communicable diseases, including the need to:

[*S*]cale up prevention, early diagnosis, and treatment of asthma and chronic obstructive pulmonary disease (COPD) by improving access to effective treatment, strengthening diagnostic services, and establishing structured programmes and services for the long-term management of chronic respiratory diseases.^3^

However, there is a significant gap between these policy statements and the current reality. Inhaled corticosteroids are available in only 52% of primary care settings in low- and middle-income countries and 36% in low-income countries.^4^ Likewise, there is limited access to diagnostic tools. Peak flow measurement is available in only 30% of low- and middle-income settings and 16% of low-income settings.^4^ Because of the shared symptoms between conditions, there is substantial misdiagnosis – for example, in one audit of primary care in South Africa, 20% of patients alternated diagnoses between asthma and COPD.^5^

In sub-Saharan Africa, asthma is a common condition, mainly affecting children, adolescents and young adults, and can have a high prevalence – for example, in Cape Town, 17% of teenagers in the age group 13–14 years have a history of asthma.^6^ In the African context, primary care providers are familiar with tuberculosis (TB) and acute respiratory infections, although the rational prescribing of antibiotics is still an issue. Beyond this, there is less familiarity with COPD, although this is also common – 13.4% of adults over 40 years have COPD.^7^ In our context, COPD is complicated by other conditions such as human immunodeficiency virus (HIV), TB and structural lung disease. Other important issues are early detection of lung cancer,^8,9^ tobacco smoking cessation and awareness of occupational causes of respiratory problems.^10^ The symptoms of lung cancer overlap with TB, and diagnosis is often delayed by a focus on TB, despite negative TB results.^8^

Respiratory conditions are also amplified by climate change through both direct and indirect effects.^11^ Direct effects of heat include airway inflammation, mucociliary dysfunction, desiccation of the mucus barrier, irritative bronchospasm and increased ventilation demand. Indirect effects of climate change include higher temperatures and more frequent heatwaves, more wildfires causing air pollution, more pollen and longer allergy seasons, and worse air quality from ozone and particulate matter. All of this exacerbates existing conditions such as asthma and COPD and increases the risk of respiratory infections. Communities in Africa are often more vulnerable due, for example, to informal and overcrowded housing that has higher indoor temperatures and no protection from air pollution. Many households also burn biomass for cooking or heating, which increases air pollution.

The IPCRG has been quite active in supporting multi-country projects in South America and Asia. For example, BreatheWell focuses on early identification and management of COPD in South America.^12^ They are looking at issues such as effective diagnosis by combining questionnaires and peak flow measurement,^13^ adaptation of pulmonary rehabilitation to the context, and approaches to effective smoking cessation. The National Institute of Health Research Global Health Research Unit on Respiratory Health (RESPIRE) has an enormous body of work in Asia addressing infectious and non-communicable respiratory diseases, climate change, as well as preventable risk factors.^14^ FRESHAIR4Life has focused on reducing tobacco smoking among adolescents in disadvantaged populations in several countries.^15^ There is a need to build similar collaborations in sub-Saharan Africa.

**FIGURE 1:**
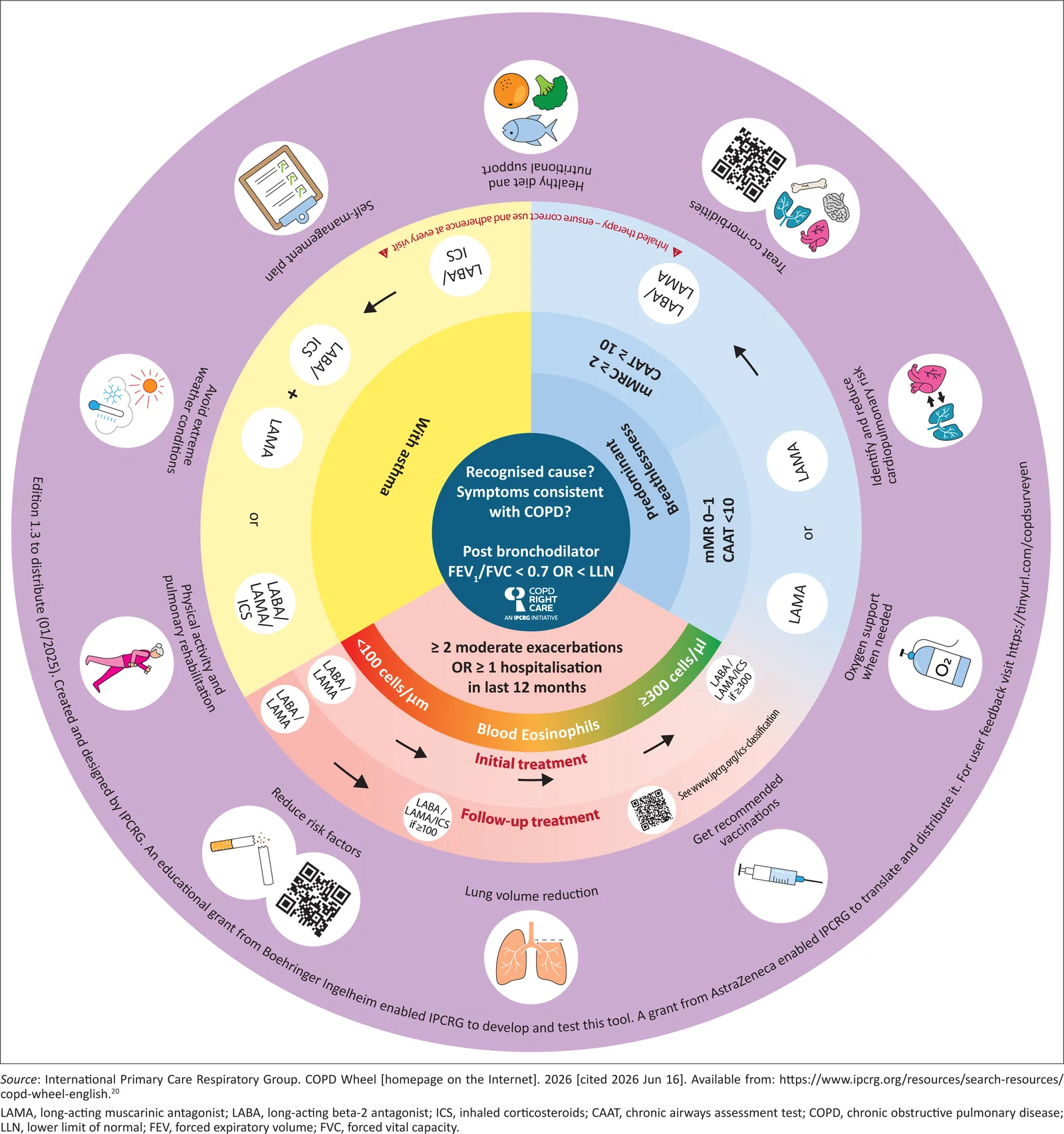
Example of the chronic obstructive pulmonary disease wheel.

Beyond these research projects, the IPCRG has also created a repository of tools and information to support primary care providers.^16^ Their desktop helper series summarises evidence for busy clinicians on diagnosis, treatment and patient education. There are opportunities for e-learning and for developing spirometry skills. The asthma and COPD Right Care campaigns provide desktop tools to support treatment decisions and education of patients, such as the COPD wheel (Figure 1). Other resources support tobacco cessation, and there are also games to use in health professions education. Consider using these resources in your education and continuous professional development (CPD) programmes.

In the African context, our focus should not be on developing and testing new medications or cutting-edge technologies, but on adapting and implementing to scale what we already know. Researchers should look to implementation research methodologies to assist with this in grant applications.^17^ Key issues to consider might be how to improve diagnostic accuracy with appropriate technology (now including artificial intelligence [AI]), deliver inhalers, provide services for smoking cessation or pulmonary rehabilitation, or share tasks between nurses, clinical officers, pharmacists and doctors within a multidisciplinary team. As always, strengthening primary care as a system is a key requirement and not just disease-specific interventions.^18^ For example, ensuring access, continuity, coordination, comprehensiveness and person-centredness.^19^

A final thought relates to the environmental determinants of health and the amplification of these risks by climate change. We need to look at how community-orientated primary care can help address issues such as heat and air pollution through local action, screening for exposure and behaviour change.^21^ We also need to embrace a primary health care approach that tackles these issues in collaboration with other sectors and the community.

In conclusion, primary care in sub-Saharan Africa should take the lead in ensuring that our patients with respiratory problems receive the ‘right care’. The volume of patients and need for ongoing care in many conditions make it essential that quality care is offered at the primary care level. However, the quality of care for conditions such as asthma and COPD in many countries and settings is not yet ‘right’. We need to advocate with our health systems to ensure the required resources are provided (e.g. inhaled corticosteroids, peak flow meters) and that our primary care providers are competent in diagnosis, treatment and prevention. Guidelines need to be adapted to the context, and implementation research helps us scale up essential interventions. Family medicine should work hand in hand with organisations such as the IPCRG to make this happen.
